# Association between polymorphism in the promoter region of lncRNA GAS5 and the risk of colorectal cancer

**DOI:** 10.1042/BSR20190091

**Published:** 2019-04-16

**Authors:** Yajie Wang, Shenshen Wu, Xi Yang, Xiaobo Li, Rui Chen

**Affiliations:** Key Laboratory of Environmental Medicine Engineering, Ministry of Education School of Public Health, Southeast University, Nanjing 210009, China

**Keywords:** colorectal cancer, GAS5, large intervening non-coding RNA, single nucleotide polymorphisms

## Abstract

The growth arrest special 5 (GAS5), as a research hotspot of long noncoding RNAs (lncRNAs), has been reported to be associated with colorectal cancer (CRC). However, the association between polymorphisms in GAS5 and the risk of CRC was not clear. In the present study, a case–control study in 1078 CRC patients and 1175 matched healthy controls was performed to evaluate the association between the potential functional genetic variants in GAS5 and the risk of CRC. PCR-TaqMan, qPCR, dual-luciferase assay, electrophoretic mobility shift assay (EMSA), flow cytometry, migration and invasion assays were performed to evaluate the function of polymorphism. Results showed that subjects carrying the rs55829688 CT/TT genotypes had a significantly higher risk of CRC when compared with the CC genotype. Further qPCR assay confirmed that the CRC tissues with rs55829688 CT/TT genotypes had a higher GAS5 mRNA expression level. The dual-luciferase assay, qPCR and EMSA assay revealed that rs55829688 T>C polymorphism could decrease the expression level of GAS5 by impacting the binding ability of the transcription factor Yin Yang-1 (YY1) to the GAS5 promoter region. The expression of apoptosis-related proteins were detected by Western blot. Further, flow cytometry, migration, and invasion experiments showed that GAS5 repressed apoptosis and increased invasion and migration capability of CRC cells. Taken together, our findings provided evidence that the rs55829688 variant in the GAS5 promoter was associated with the risk of CRC and decreased expression of GAS5 by affecting the binding affinity of the transcription factors YY1 to GAS5.

## Introduction

Colorectal cancer (CRC) is one of the most common types of cancer worldwide, with 1.36 million new cases of CRC and 694000 deaths in 2012 [1]. The current evidence points to the increasing rate of CRC in young adults less than 50 years [2] and some environmental factors, including smoking [3], drinking [4,5], and diet [6,7] can affect the risk of CRC. Genetic and epigenetic factors were also associated with CRC [8–10]. However, in spite of great effort for the research of CRC carcinogenesis, the etiology of CRC is not completely clear at present.

As the research hotspot of long noncoding RNAs (lncRNAs), GAS5 was initially found to be highly expressed in mouse NIH3T3 fibroblasts with growth inhibition [11]. In recent years, studies have shown that GAS5 plays a significant role in the development of tumors including breast cancer [12], cervical cancer [13], prostate cancer [14] etc. Single nucleotide polymorphism (SNP) is the DNA sequence polymorphism caused by single nucleotide variation at the genomic level. It is the most common form of human genetic variation, accounting for more than 90% of all known polymorphisms [15]. Previous studies have shown that GAS5 polymorphisms are associated with multiple diseases [16–18]; however, the association and mechanism between GAS5 and the risk of CRC remains unclear.

Therefore, a case–control study on 1078 CRC patients and 1175 matched cancer-free controls was conducted to evaluate the association of the selected genetic variants in GAS5 with the risk of CRC, and further reveal the functional mechanisms of CRC risk.

## Materials and methods

### Study subjects

A total of 1078 patients with CRC and 1175 cancer-free controls was included in this case–control study as previously reported [19]. Briefly, patients with CRC were newly diagnosed and histologically confirmed from hospitals in Jiangsu province between January 2007 and October 2011. Additionally, patients with history of organ transplantation and other cancers were excluded. And controls were randomly selected from individuals who attended the physical examination in the same district. Subjects were determined to be no CRC and had no biological association with the case group after the physical examination. The frequency of age and gender were matched with subjects in the case group, and the difference was not statistically significant. The tumor tissues from patients with CRC were collected after surgical resection. Peripheral blood was collected from controls after informed consent was obtained from each participant. All the enrolled subjects signed the informed consent before recruitment. The Institutional Review Board (IRB) of Southeast University approved the present study.

### SNP selection and genotype analysis

The specific position information of GAS5 on the chromosome was obtained from the 1000 Genomes database. The linkage disequilibrium (LD) analysis of the *GAS5* gene was performed using a VCF-PED converter and Haploview 4.2 software. TagSNPs were selected with the minimum allele frequency (MAF) of the Han Chinese in Beijing, China (CHB) ≥ 5% and the threshold r^2^ between SNPs in the gene haplotype ≥ 0.8. Combined with the position information of SNPs in GAS5 obtained from UCSC and NCBI, the final SNPs were selected. EZNA Tissue DNA Kit (Omega Bio-Tek, Norcross, U.S.A.) was used for extraction of the genomic DNA of CRC patients that from paraffin-embedded CRC tissues. RelaxGene Blood DNA System (Tiangen Biotech, Beijing, China) was used for extraction of the genomic DNA of controls that from peripheral blood. TaqMan allelic discrimination assays were used to detect the genotypes of selected polymorphisms with the Quant Studio 6 Flex system (Applied Biosystems, CA, U.S.A.). Approximately 10% percent of the samples weres randomly selected to repeat the genotyping results. The concordance rate was 100%.

### Real-time PCR analysis of GAS5

Total RNAs used in the present study were extracted from the tissues using the TRIzol reagent (Invitrogen, Carlsbad, CA). The cDNA synthesis was performed with 1 μg of total RNA according to the manufacturer’s protocol (Takara, Tokyo, Japan). GAPDH (forward, 5′-TCGGAGTCAACGGATTTGG-3′ and reverse, 5′-CATGGGTGGAATCATATTGGA-3′) was used as internal control in SYBR Green Real-time PCR Master Mix-Plus kits (Toyobo, Osaka, Japan). The relative RNA levels of GAS5 were determined with the Quant Studio 6 Flex system (Applied Biosystems, Life Technologies, U.S.A.). The relative quantitative value was shown by the 2^−ΔΔCt^. The experiment was carried out in triplicates.

### Cell culture

We purchased the human CRC cells (HT29, SW480, HCT15, RKO, LOVO, and DLD-1) from the Shanghai Institute of Biochemistry and Cell Biology, Chinese Academy of Sciences (Shanghai, China), and maintained in Dulbecco’s modified Eagle’s medium (DMEM, HyClone, UT, U.S.A.) in 5% CO_2_ at 37°C. Culture medium was supplemented with 10% fetal bovine serum (FBS) (Sigma, MO, U.S.A.), 100 μg/ml streptomycin and 100 U/ml penicillin (HyClone, UT, U.S.A.). The cells were tested and validated to be free of mycoplasma.

### Luciferase reporter assay

The GAS5 promoter sequence containing rs55829688 T or C allele was constructed into pGL3-basic vector (pGL3-basic-GAS5-WT or pGL3-basic-GAS5-MT). CRC cells were planted at a density of 3 × 10^5^ cells/well in six-well plates. Twenty-four hours later, cells were transfected by Lipofectamine 2000 (Invitrogen, CA, U.S.A.) according to the manufacturer’s protocol. In each well, 1 μg of reconstructed vector pGL3-basic-GAS5-WT or pGL3-basic-GAS5-MT and 1 μg of pRL-SV40 (Promega, Madison, U.S.A.) were co-transfected. Forty-eight hours after transfection, cells were harvested immediately and Renilla/Firefly luciferase activity in cell lysate was measured with the Dual-Luciferase Reporter Assay System (Promega, Madison, U.S.A.).

### Electrophoretic mobility shift assay

For electrophoretic mobility shift assay (EMSA), nuclear extracts were isolated from CRC cells with NEPER Nuclear and Cytoplasmic Extraction Reagents (Thermo Fisher Scientific, Shanghai, China) according to the manufacturer’s instructions. Biotin-labeled RNA probes were obtained by in vitro transcription assays with Biotin RNA Labeling Mix (Roche, Basel, Switzerland). After incubation in 1× EMSA binding buffer supplemented with glycerol, KCl, and MgCl_2_ for 30 min according to the manufacturer’s instructions for the LightShift Chemiluminescent RNA EMSA Kit (Thermo Fisher Scientific, Shanghai, China), the components were separated with native PAGE and then transferred on to positively charged NC film (Beyotime, Shanghai, China). After UV cross-linking, biotin signals were detected with HRP–conjugated streptavidin according to the manufacturer’s instructions for the Chemiluminescent Nucleic Acid Detection Module (Thermo Fisher Scientific, Shanghai, China).

### siRNA transfection

GAS5 siRNAs (three siRNA sequences (5′–3′) were as follows: siRNA1: GCAAGCCTAACTCAAGCCA; siRNA2: GGACCAGCTTAATGGTTCT; siRNA3: GCAAAGGACTCAGAATTCA) were chemically synthesized (Ruibo, Guangzhou, China). The CRC cells were transfected with siRNA in accordance with the manufacturer’s instructions. Briefly, the original stock of the siRNA was diluted to 20 μM by RNase-free water and stored at −20°C until use. A total of 3 × 10^5^ cells were seeded in six-well plates 24 h before the transfection procedure. Two groups were separately transfected with 5 μl negative control siRNA (NC) and GAS5 siRNA using Lipofectamine 2000 reagent (Invitrogen, Carlsbad, CA) according to the manufacturer’s protocol. Cells were collected 48 h after transfection.

### Cell proliferation assay

Cell viability was measured with the Cell Counting Kit-8 (Sigma, Darmstadt, Germany) according to the manufacturer’s instructions. A total of 5 × 10^3^ transfected CRC cells were plated into each well of a 96-well plate with DMEM containing 10% FBS. CCK-8 was added and cells were arrested every 24 h, the mixture was incubated at 37°C for 2 h. The absorbance at 450 nm (OD = 450 nm) was detected with a microplate reader.

### In vitro migration and invasion assays

For the cell invasion assays, after 48 h of transfection, 5 × 10^4^ CRC cells were plated at the top of the Matrigel-coated invasion chambers (CytoSelect 24-Well Cell Invasion Assay Kit; Cell Biolabs, U.S.A.) with a serum-free DMEM in six-well plates; 10% FBS DMEM was added to the lower chamber and cultured for 24 h. For cell migration assays, after 48 h of transfection, 5 × 10^4^ cells were plated into the transwell chamber, and cultured with complete medium for 24 h. Finally, all cells were fixed with ethanol and stained with Crystal Violet.

### Flow cytometry

A total of 5 × 10^5^ CRC cells were seeded into six-well plate one day before transfection and cells were harvested 48 h after transfection. Apoptotic cells were stained with Annexin V-FITC/PI (BD, CA, U.S.A.) at room temperature in the dark. The treated cells were analyzed via FACSCanto™ II flow cytometer (BD, CA, U.S.A.). Cell populations were classified as viable (Annexin V negative, PI negative), early apoptotic (Annexin V positive, PI negative), late apoptotic (Annexin V positive, PI positive), or necrotic (Annexin V negative, PI positive). For cell-cycle analysis, cells were fixed in 100% ethanol and stained with propidium iodide and RNase A (KeyGen Biotech, Nanjing, China). All assays were independently performed in triplicates.

### Western blot

Protein concentrations were determined by Pierce™ BCA Protein Assay Kit (Thermo Fisher Scientific, Shanghai, China). Approximately 10 μg of cells protein lysates were analyzed by Western blot. Western blot was performed by the standard protocols. Primary antibodies against cleaved caspase 3, cleaved PARP (Cell Signaling Technology, Danvers, U.S.A.), β-actin (CMCTAG, Shanghai, China), and HRP–conjugated secondary antibodies (Cell Signaling Technology, Danvers, U.S.A.) were used.

### Statistical methods

Statistical analyses were performed by SAS 9.4 (SAS Institute, Cary, NC). The goodness-of-fit *χ^2^* test (*χ^2^* test) was used to estimate the Hardy–Weinberg equilibrium (HWE) among the controls. The *χ^2^* test and *t* test were used to evaluate the difference in clinical pathological features. The association between the genotypes and risk of CRC were estimated by odds ratios (ORs) and 95% confidence intervals (CIs). Logistic regression was used to analyze the association among rs55829688, rs1951625, and CRC risk, adjusted by gender and age. Student’s t test was used to examine the difference of the luciferase activity. All the tests were two-sided. The statistical significance criterion was set at *P* < 0.05.

## Results

### Characteristics of research subjects

The demographic and clinical detailed information of the CRC cases and controls has been summarized previously [20]. The distributions of age and sex of the cases and controls were similar. The frequencies of colon cancer in cases were 44.1% and the frequencies of rectal cancer in cases were 55.9%. The frequencies of distant metastasis of M0 and M1 were 88.9 and 11.1%. Furthermore, the frequencies of the lymph node metastasis of N0 and N1 were 57.6 and 42.4%.

### Association between the GAS5 polymorphisms and CRC susceptibility

As shown in Table 1, all the observed genotype frequencies of the three polymorphisms (rs55829688 and rs1951625) in the controls were in agreement with the HWE (*P* = 0.5921 for rs55829688, *P* = 0.5226 for rs1951625). The results demonstrated significant association between rs55829688 genotype distributions and the risk of CRC after adjusting age and gender (CT vs. CC, adjusted OR = 1.50, 95% CI = 1.10–2.03; TT vs. CC, adjusted OR = 1.64, 95% CI = 1.21–2.22, respectively). The recessive genetic model showed a significantly increased risk of CRC in patients with CT/TT after adjusting for age and gender (CT/TT vs. CC, adjusted OR = 1.57, 95% CI = 1.17–2.10). In addition, stratified analysis showed the increased risk in each subgroup of age ≤ 56 years and females. We also found that the rs55829688 CT/TT genotypes were associated with the increased risk of both colon and rectum cancer and low grade of CRC (Table 2).

**Table 1:** Association between GAS5 polymorphisms and CRC risk

Genotype	Case^2^		Control^2^		*P*	Adjusted OR (95% CI)^1^
	*n*	%	*N*	%		
rs55829688						
CC	81	7.7	130	11.4	0.0066	
CT	460	43.5	500	43.9		1.50 (1.10–2.03)
TT	516	48.8	509	44.7		1.64 (1.21–2.22)
CT/TT	976	92.3	1009	88.6		1.57 (1.17–2.10)
T allele	0.6953		0.6919		0.0062	
HWE	0.3752		0.5921			
*P* trend					0.0016	
rs1951625						
CC	490	45.5	524	44.6	0.8853	
CT	468	43.4	514	43.7		0.97 (0.82–1.16)
TT	120	11.1	137	11.7		0.94 (0.71–1.23)
T allele	0.3284		0.3353		0.6215	
HWE	0.6044		0.5226			
*P* trend					0.6245	

1 Adjusted for age and gender in the logistic regression model.

2 The mismatch between the number of genotyping samples and a total of samples is due to the absence of samples.

**Table 2:** Stratified analysis of GAS5 rs55829688 genotypes associated with CRC risk

Variables	Genotypes (cases/controls)		*P*	Adjusted HR
	CC	CT/TT	(95% CI)^1^	
Age (years)				
≤56	44/69	516/507	0.0203	1.57 (1.05–2.35)
>56	37/61	460/502	0.0572	1.52 (0.99–240)
Gender				
Male	57/79	591/579	0.0576	1.39 (0.97–2.00)
Female	24/51	385/430	0.0113	1.93 (1.16–3.21)
Location				
Colon	35/130	432/1009	0.0188	1.61 (1.08–2.40)
Rectum	46/130	544/1009	0.0184	1.55 (1.09–2.21)
Grade				
Low	18/130	330/1009	0.0007	2.32 (1.39–3.87)
Intermediate/High	63/130	646/1009	0.0840	1.34 (0.97–1.84)
Depth of invasion				
T1	3/130	32/1009	0.8876	1.99 (1.28–3.08)
T2	28/130	212/1009	0.9812	1.58 (1.10–2.28)
T3	20/130	178/1009	0.6013	1.43 (0.99–2.05)
T4	30/130	554/1009	<0.0001	1.11 (0.72–1.69)
Lymph node metastasis				
N0	46/130	562/1009	0.0109	1.58 (1.11–2.25)
N1	35/130	414/1009	0.0333	1.54 (1.04–2.27)
Distant metastasis				
M0	70/130	870/1009	0.0023	1.62 (1.20–2.20)
M1	11/130	106/1009	0.5118	1.24 (0.65–2.37)
TNM				
I	20/130	174/1009	0.6528	1.11 (0.67–1.82)
II	24/130	366/1009	0.0097	2.01 (1.28–3.16)
III	26/130	330/1009	0.0268	1.65 (1.06–2.57)
IV	11/130	106/1009	0.5115	1.24 (0.65–2.37)

1 Adjusted for age, gender in the logistic regression model.

### The correlation between rs55829688 and GAS5 expression

**Figure 1:**
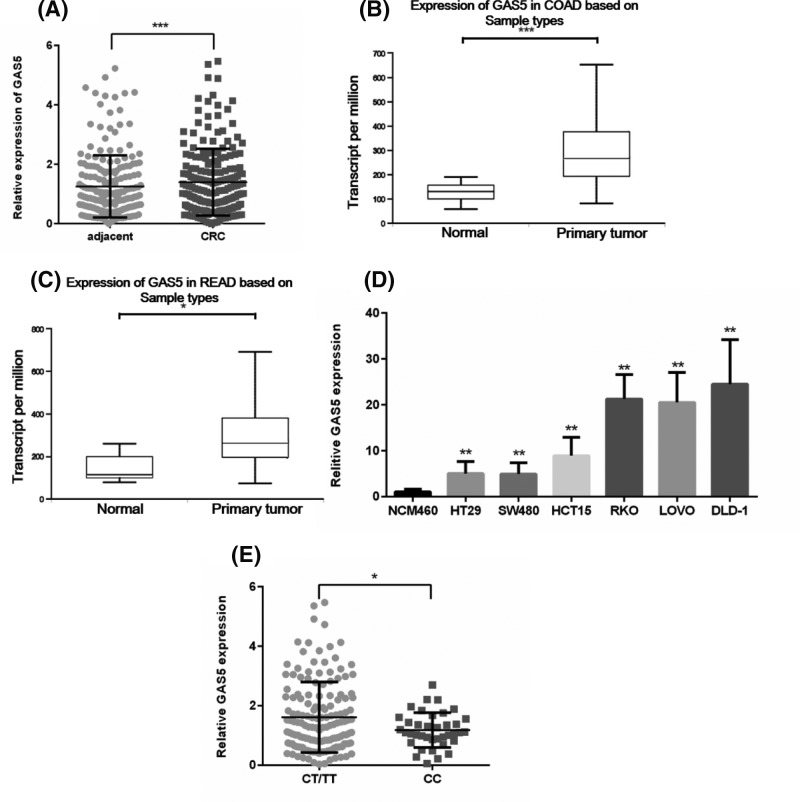
GAS5 is overexpressed in human CRC (**A**) GAS5 expression was determined by qRT-PCR in 210 paired CRC samples and adjacent normal tissues. (**B,C**) Analysis of GAS5 expression in CRC in TCGA database. (**B**) Expression of GAS5 in COAD (COAD, Colon adenoma; Normal n=41, Primary tumor n=286, ***P<0.001). (**C**) Expression of GAS5 in READ (READ, Rectal adenoma; Normal n=10, Primary tumor n=166, *P<0.05). (**D**) The expression levels of GAS5 were determined by qPCR in human normal colorectal mucosa cell NCM460 and CRC cell lines (**P<0.01). (**E**) GAS5 mRNA levels in CRC tissues expressing CT/TT or CC genotype were detected by qPCR analysis (*P<0.05). CT/TT, n=164, CC, n=46.

We measured the expression of GAS5 in CRC samples and adjacent normal tissues. Result showed that GAS5 was significantly up-regulated in CRC tissues compared with paired normal tissues (Figure 1A). Next, we analyzed GAS5 expression in CRC samples in The Cancer Genome Atlas (TCGA) database. In colorectal adenoma panel, the number of normal tissues is 41 and the number of primary tumors is 286 (Figure 1B). In rectal adenoma panel, the number of normal tissue is 10 and the number of primary tumor is 166 (Figure 1C). It indicated that compared with normal tissues, GAS5 was highly expressed in both colon adenomas and rectal adenomas (*P* < 0.001 and *P* < 0.05). Next, we examined the expression of GAS5 in CRC cells. Similar to the result of GAS5 expression in CRC tissues, the expression of GAS5 was also higher in CRC cell lines (HT29, SW480, HCT15, RKO, LOVO, and DLD-1) than that in human normal colorectal mucosa cell NCM460 (Figure 1D). Then we analyzed the GAS5 mRNA level in CRC tissue samples with different genotypes. The relative GAS5 expression level in samples with rs55829688 CT/TT genotype was significantly higher than that in samples with CC genotype (Figure 1E, *P* < 0.05).

### Rs55829688 CT/TT genotype increased the binding affinity of Yin Yang-1 to the GAS5 promoter

**Figure 2:**
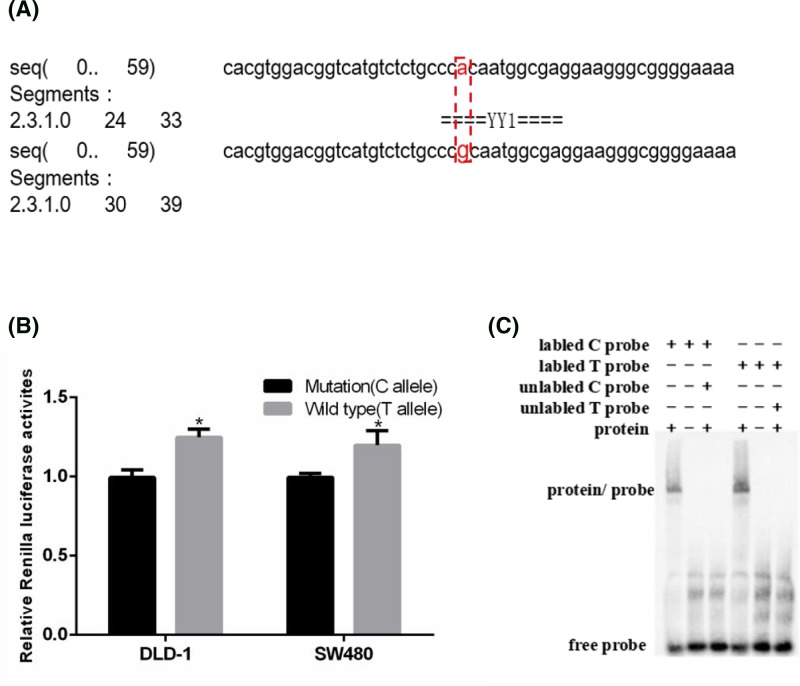
rs55829688 T>C genotype decreases the binding affinity of YY1 to the GAS5 promoter (**A**) Prediction of the binding affinity of YY1 to the mutation region of rs55829688 with the bioinformatics algorithm (AliBaba2). (**B**) The effect of rs55829688 on GAS5 transcriptional activity as determined by luciferase reporter assay. The luciferase activity of the cells transfected with pGL3-basic-GAS5-WT was higher than that of DLD-1 and SW480 cells transfected with pGL3-basic-GAS5-MT (*P<0.05). (**C**) EMSA showed that effect of GAS5 gene promoter region rs55829688 T>C polymorphism on the binding of GAS5 gene promoter region and transcription factor YY1.

Given that the SNP rs55829688 T>C polymorphism was located in the promoter region of GAS5, we then analyzed the promoter region surrounding it using a bioinformatics algorithm (AliBaba2). The result showed that the rs55829688 T>C polymorphism may change the binding affinity of Yin Yang-1 (YY1) to the rs55829688 mutation region (Figure 2A). To further explore whether rs55829688 polymorphism can affect the transcriptional activity of GAS5, dual-luciferase reporter assay was performed. Result showed that DLD-1 and SW480 cells transfected with the plasmid carrying T allele exhibited the significantly higher luciferase activity than that with C allele (Figure 2B, *P* < 0.05). To determine whether rs55829688 T>C polymorphism could affect the different binding ability on transcription factors, EMSA experiment was performed with biotin-labeled probes containing either T or C allele. Results revealed compared with rs55829688 C allele probe, the rs55829688 T allele probe showed greater binding of protein or complex in cells (Figure 2C). Taken together, these data indicate a possible effect of the GAS5 promoter polymorphism on GAS5 transcription activity.

### GAS5 knockdown promotes cell apoptosis

**Figure 3:**
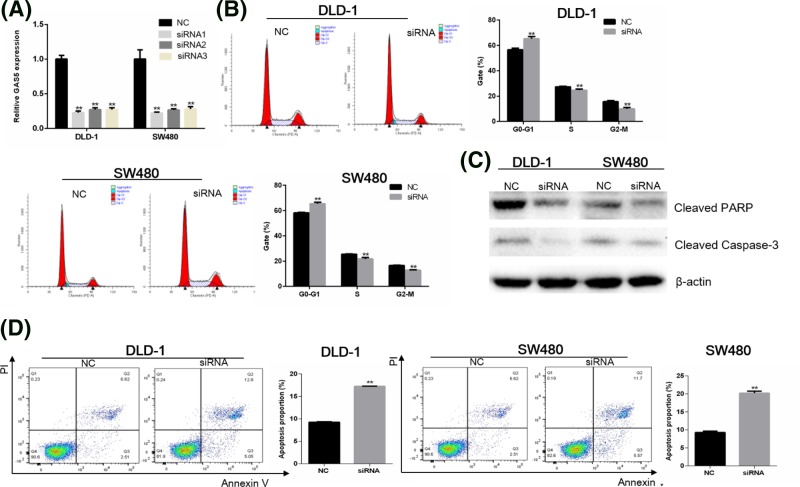
GAS5 knockdown induces the apoptosis of DLD-1 and SW480 cells (**A**) GAS5 mRNA expression following transfection with GAS5 siRNA in DLD-1 and SW480 cells compared with transfection with the NC (the efficiency of three siRNAs in DLD-1 cells was 77.06, 72.71, and 72.50% respectively, **P<0.01. The efficiency of three siRNAs in SW480 cells was 76.34, 72.59, and 71.46% respectively, **P<0.01). (**B**) Flow cytometric analysis of cell-cycle profiles (**P<0.01, compared with NC control). (**C**) GAS5 knockdown decreased the expression of cleaved PARP and cleaved caspase 3 in DLD-1 and SW480 cells. (**D**) Flow cytometric analysis of apoptosis. Cells were stained with Annexin/PI. Values were presented as mean ± standard (**P<0.01, compared with NC control).

To investigate the effect of GAS5 on apoptosis, knockdown of GAS5 with siRNA in DLD-1 and SW480 cells were performed. The efficiency of three siRNAs in DLD-1 cells was 77.06, 72.71, and 72.50% respectively, and the efficiency of three siRNAs in SW480 cells was 76.34, 72.59, and 71.46% respectively (Figure 3A, *P* < 0.01). The most effective siRNAs were selected for knockdown experiments. Flow cytometry analysis was performed to determine the effect of GAS5 on cell cycle and apoptosis of CRC cells. Compared with NC group, a delay of G_0_/G_1_ to S-phase transition were observed in siRNA group (Figure 3B). Western blot was then performed to evaluate whether GAS5 regulates apoptosis-related protein in CRC cells. Results demonstrated that knockdown of GAS5 could decrease protein level of cleaved PARP and cleaved caspase 3 (Figure 3C). Finally, by staining with Annexin V/PI, we found that the rate of apoptosis was significantly increased following knockdown of GAS5 (Figure 3D). These results indicated that GAS5 expression was an important determinant of apoptosis in DLD-1 and SW480 cells, and supported the opinion that GAS5 acted as a tumor promoter in CRC through restraining apoptosis and regulating early cell cycle.

### Analysis of cell proliferation, invasion and migration ability

**Figure 4:**
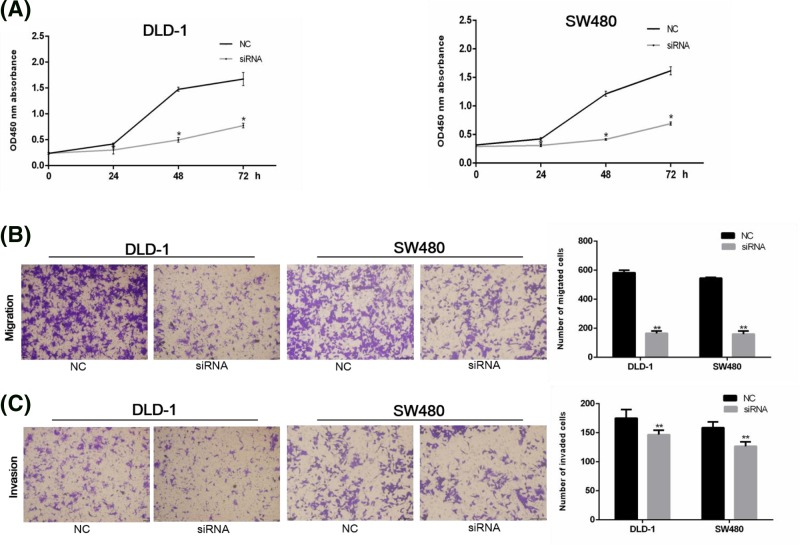
GAS5 knockdown inhibits DLD-1 and SW480 cells proliferation, migration, and invasion (**A**) DLD-1 and SW480 cells treated by GAS5 siRNA show lower OD values 0, 24, 48, 72 h after CCK-8 treatment (*P<0.05). (**B**-**C**) DLD-1 and SW480 cells migration and invasion capacity in both NC and siRNA transfected cells. Compared with NC group, cells transfected with siRNA had a lower ability of migration and invasion (**P<0.01). Results were shown as the mean ± standard.

To further investigate the effect of GAS5 on cell proliferation, invasion and migration ability, we examined cell proliferation ability by CCK-8 assay. Compared with the NC group, the growth of DLD-1 and SW480 cells was slower in siRNA group (*P* < 0.05) at 24h, 48h, and 72h time periods (Figure 4A). Further migration and invasion assays showed that compared with NC group, siRNA group had a lower ability of migration and invasion (Figure 4B,C). These results suggested that elevated GAS5 expression can increase the ability of cell proliferation, invasion, and migration.

## Discussion

In our study, we found that subjects with the rs55829688 CT/TT genotypes had a significantly increased risk when compared with those with the CC genotype. Further functional study revealed that the rs55829688 T>C polymorphism may change the binding affinity of the transcription factors YY1 to the rs55829688 mutation region, leading to lower expression of GAS5, and ultimately repress the development of CRC. Furthermore, migration, invasion and flow cytometry demonstrated GAS5 inhibited apoptosis and promoted proliferation, invasion, and migration ability of CRC cells.

Increasing data showed that abnormal expression of GAS5 is involved in many kinds of cancers and its aberrant expression is associated with lung cancer [21], renal cell carcinoma [22], breast cancer [12], glioblastoma [23], and CRC [24]. Meanwhile, the expression level of GAS5 was correlated with tumor size, histological grade, and TNM stage in CRC [11]. However, opinions about the role of GAS5 in CRC were different. Most studies reported that GAS5 played a tumor suppressor role in CRC [11,24–28]. On the contrary, Song found GAS5 participates in the occurrence of CRC [29], but there was no further mechanism study about it.

Previous studies have revealed that polymorphisms in the promoter region of GAS5 are associated with different types of cancers [30,31]. For example, Li et al. [18] found that rs145204276 in the promoter region of GAS5 played a protective role in the development of gastric cancer through the regulation of GAS5. Tao et al. [17] found that rs145204276 might affect the expression level of GAS5 by disturbing its transcript activity and consequently increased with risk of hepatocellular carcinoma. Recently, Zheng et al. [32] found genetic variation rs145204276 of GAS5 can affect the development and metastasis process of CRC in a Chinese population. The allele del of rs145204276 was associated with the decreased risk of CRC [32], but the mechanism study was not performed. In the present study, we confirmed that rs55829688 in the promoter region of GAS5 was associated with the risk of CRC. Moreover, rs55829688 T>C polymorphism changed the expression level of GAS5 through alter the transcription activity of GAS5 and further influenced the development of CRC. These results suggested that GAS5 polymorphism played an important role in the development of CRC.

In our study, we found that GAS5 played a role in cancer promotion in CRC. Although our finding existed a departure from the literature, our procedures were highly rigorous. By contrast, we have taken into account several critical considerations in our study. First, we found that compared with normal tissues, GAS5 was up-regulated in CRC tissues. TCGA database indicated that compared with normal tissues, GAS5 was highly expressed in both colon adenomas and rectal adenomas. Our results were consistent with that of TCGA database, the most commonly and largest used public data resource, which consisted of gene expression and molecular data from a large amount of tumor samples [33]. And our results were also similar to the reports of Song that were mentioned previously [29]. Second, compared with other studies related to CRC and GAS5 expression [24–26], the number of paired cancer and adjacent tissues that we used to detect GAS5 expression differences was 210 pairs. The amount of samples was relatively large and more convincing. Third, we have carried out systematic verification throughout the experimental design, and obtained corresponding data and results. Mechanisms about the risk of GAS5 on CRC is complex, different population, samples and environments might have influence on it. Therefore, further functional researches are required to verify our results.

Finally, limitations in our study should be mentioned. First, further verification of GAS5 expression in CRC including more population is still required. Second, in our study, GAS5 was overexpressed in CRC, we primarily performed the knockdown experiment and verified the biological effect of GAS5. It would be more complete and rigorous if we performed experiment about overexpression of GAS5 or mutation GAS5.

In conclusion, we reported that rs55829688 T>C polymorphism could alter the transcription activity of GAS5 gene, thereby changing the expression level of GAS5 and further influencing the development of CRC.

## Abbreviations

CCK-8: Cell Counting Kit-8
CI: confidence interval
CRC: colorectal cancer
DMEM: Dulbecco’s modified Eagle’s medium
EMSA: electrophoretic mobility shift assay
FBS: fetal bovine serum
GAPDH: glyceraldehyde-3-phosphate dehydrogenase
GAS5: growth arrest special 5
HRP: horse radish peroxidase
HWE: Hardy–Weinberg equilibrium
NC: negative control siRNA
OD: optical density
OR: odds ratio
PARP: poly ADP-ribose polymerase
qPCR: Real-time Quantitative PCR
SNP: single nucleotide polymorphism
TCGA: The Cancer Genome Atlas
TNM: tumour node metastases
VCF-PED: VCF to PED converter
YY1: Yin Yang-1
