# Correction: Association between polymorphism in the promoter region of lncRNA GAS5 and the risk of colorectal cancer

**DOI:** 10.1042/BSR-20190091_COR

**Published:** 2019-05-21

**Bioscience Reports (2019) 39(4), BSR20190091; https://doi.org/10.1042/BSR20190091**

The authors have noticed an error in their published article. In the section **GAS5 knockdown promotes cell apoptosis**, “Results demonstrated that knockdown of GAS5 could **decrease** protein level of cleaved PARP and cleaved caspase 3 (Figure 3C).” should read “Results demonstrated that knockdown of GAS5 could **increase** protein level of cleaved PARP and cleaved caspase 3 (Figure 3C).”

Furthermore, in the Figure 3 legend “GAS5 knockdown **decreased** the expression of cleaved PARP and cleaved caspase 3 in DLD-1 and SW480 cells.” should read “GAS5 knockdown **increased** the expression of cleaved PARP and cleaved caspase 3 in DLD-1 and SW480 cells.”

